# Pancreas Injury and Abdominal Pain as a Result of Duodenal Perforation by an Ingested Toothpick

**DOI:** 10.14309/crj.0000000000001602

**Published:** 2025-02-05

**Authors:** Jia-ju Li, Lei Wang

**Affiliations:** 1 Department of Gastroenterology and Hepatology, People's Hospital of Weining Yi, Hui and Miao Autonomous County, Guizhou, China

## CASE REPORT

A 37-year-old man accidently swallowed a hard object while intoxicated 3 weeks ago. He initially felt mild throat discomfort but ignored it. Since then, he has experienced occasional abdominal discomfort. One week ago, the patient went to our hospital due to mild upper abdominal pain. The physical examination presented negative sign.

**Figure 1.**
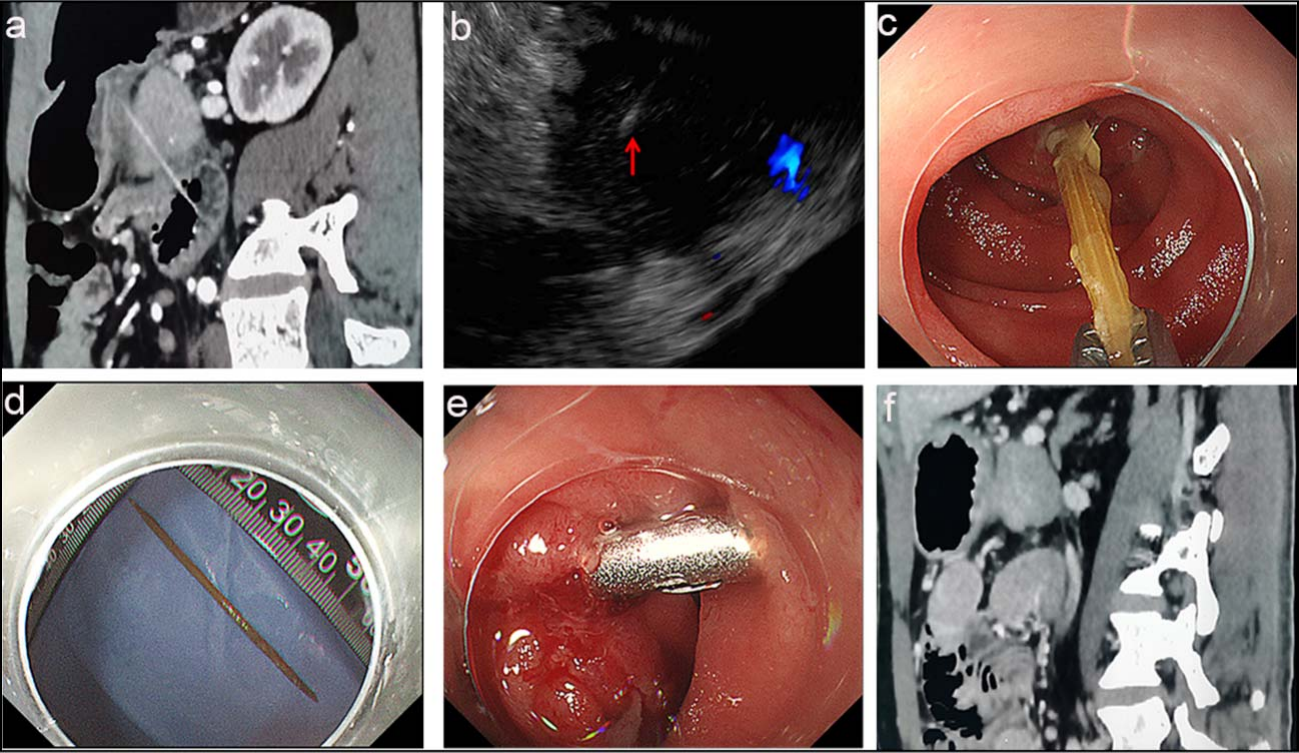
CE-CT indicated that a 6-cm foreign body traversing from the pancreas head to the small intestine (a). The red arrow indicates a long, thin, hyperechoic foreign body crossing the pancreas as confirmed by endoscopic ultrasonography (EUS) (b).The toothpick was removed under enteroscopy using foreign body forceps (c). The toothpick image (d). The postoperative wound was completely closed by endoscopic clips (e). CE-CT 3 days later showed no significant complications (f).

The patient went to our department 1 day ago, and underwent an abdominal contrast-enhanced computed tomography (CE-CT). The images of CE-CT indicated that a 6-cm foreign body traversing from the pancreas head to the small intestine (Figure 1). Further endoscopic ultrasound confirmed that a long and thin hyperechogenic foreign body penetrated through the pancreas, and fortunately, no obvious injury of blood vessels, pancreatic, or biliary duct was found (Figure 1). Thus, we planned to remove the foreign body under endoscopy. Under enteroscopy, a 3-cm foreign body was found penetrating the intestinal wall about 15-cm away from the duodenal papilla (Figure 1). The toothpick was successfully extracted using foreign body forceps under transparent cap-assisted enteroscopy (Figure 1). As a small perforation and few necrotic tissues were seen, the postoperative wound was completely closed by endoscopic clips (Figure 1) and nasogastric tube drainage was conducted for 3 days. Combined with somatostatin and antibiotics administration for 3 days, the patient's abdominal pain was significantly improved and discharged from hospital without severe complications (Figure 1).

When the patient consumed alcohol, he entered an inebriated state and was unable to recall the hard object he had ingested. Consequently, the identification of the foreign body using standard CT imaging proved challenging, and gastroscopy was limited to accessing only the posterior wall of the duodenal bulb. As a result, such cases were frequently overlooked by doctors. Therefore, we need to find more detailed tests, such as CE-CT and small bowel endoscopy. It is safe to use enteroscopy to remove small intestinal foreign bodies-penetrating pancreatic foreign bodies without pancreatic duct and blood vessel injury. Surgical treatment was avoided.

## DISCLOSURES

Author contributions: J. Li collected the cases and wrote the manuscript. W. Lei reviewed and revised the manuscript. W. Lei is the article guarantor.

Financial disclosure: None to report.

Informed consent was obtained for this case report.

